# Hormone Replacement Therapy in Females Can Decrease the Risk of Lung Cancer: A Meta-Analysis

**DOI:** 10.1371/journal.pone.0071236

**Published:** 2013-08-14

**Authors:** Yanwen Yao, Xiaoling Gu, Juehua Zhu, Dongmei Yuan, Yong Song

**Affiliations:** 1 Jinling Hospital, Department of Respiratory Medicine, Nanjing University, School of Medicine, Nanjing, China; 2 Jinling Hospital, Department of Neurology Medicine, Nanjing University, School of Medicine, Nanjing, China; National Taiwan University, Taiwan

## Abstract

The purpose of the present meta-analysis was to determine the relationship between hormone replacement therapy (HRT) and lung cancer risk in females. Publications were reviewed and obtained through a PubMed, EMBASE database and Cochrane Library literature search up to May, 2012. The detailed numbers of patients in different groups, odd ratios (ORs) and corresponding 95% confidence intervals (CIs) were collected and estimated using a random-effects model. Twenty five studies entered into the meta-analysis. The total number of participates and lung cancer patients was 656,403 and 11,442, respectively. The OR of all 25 studies was 0.91 (95%CI  = 0.83 to 0.99) and P value was 0.033. In stratified analyses, the positive association between HRT use and decreased lung cancer risk was also found in the patients with BMI<25 kg/m^2^ (OR = 0.65, P = 0.000), and never smokers patients (OR = 0.86, P = 0.042). However, HRT use in patients with artificial menopause could increase the lung cancer risk, OR = 1.51(P = 0.001). The result of Egger's test did not show any evidence of publican bias (P = 0.069). In conclusion, our meta-analysis on HRT and lung cancer risk suggests that HRT use is correlated with decreased lung cancer risk in female, especially in female with BMI<25 kg/m^2^ and never smokers.

## Introduction

Lung cancer is now the most commonly diagnosed cancer and the leading cause of cancer mortality in both men and women worldwide. Since less females smoke than males or females started smoking later than males, lung cancer trends among females lag behind males [1]. However, lung cancer incidence still keeps increasing [2]. Besides smoking, several occupational and environmental carcinogens such as asbestos, arsenic and radon are known to be risk factors for lung cancer in females [1]. Hormone replacement therapy (HRT) remains the most effective treatment for postmenopausal symptoms in menopausal females and young females who go into early menopause due to surgery or chemotherapy or radiotherapy to the pelvic region [3]. Adami et al. first demonstrated that lung cancer risk increased in women receiving HRT [4]. During the past two decades, studies on the HRT and lung cancer risk have demonstrated contradictory results, making the association still remained uncertain. Two clinical trials from Chlebowski confirmed the correlation between increased lung cancer risk and HRT [5], [6], but Chen et al., Clague et al. and Ramnath et al. showed in their studies that HRT use in females can decrease the incidence of lung cancer [7]–[9].

In order to confirm a definite correlation between HRT and lung cancer risk, we conducted a literature search and performed meta-analysis of available studies.

## Materials and Methods

### Literature search and identification of the publications

**Figure 1:**
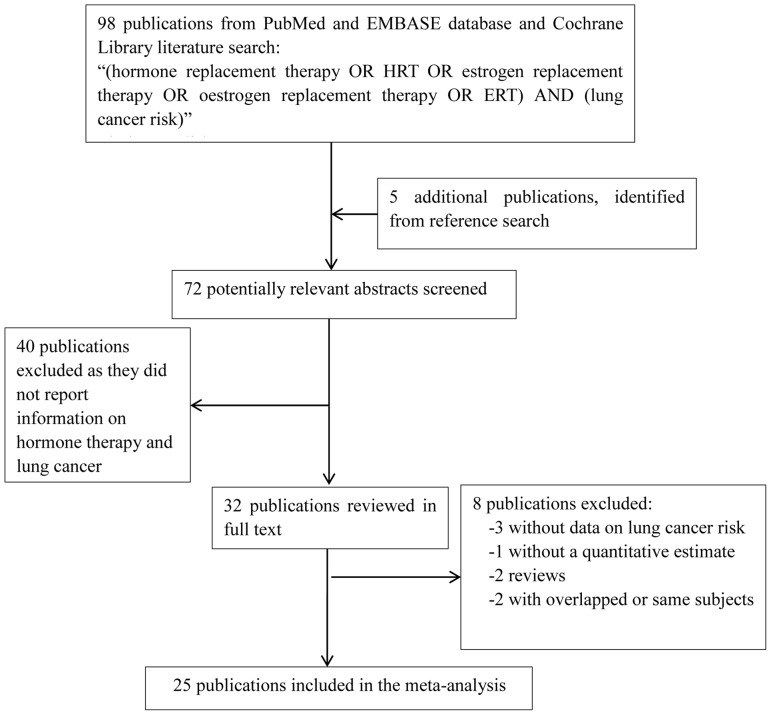
Flowchart of study identifying and including studies.

A literature search of the PubMed, EMBASE databases and Cochrane Library (updated to May, 2012) was conducted using combinations of the following terms: “hormone replacement therapy”, “HRT”, “oestrogen replacement therapy”, “estrogen replacement therapy”, “progestin replacement therapy”, “ERT”, “lung cancer”. We limited the search to studies in human and written in English. Two of the authors (Yanwen Yao and Xiaoling Gu) independently selected the articles with information on the association between HRT and lung cancer morbidity, and further check the reference list of the publications. All studies providing estimates of odd ratio (OR) or relative risk (RR) or hazard ratio (HR) and the corresponding 95% confidence interval (CI), or information sufficient to calculate them, were included in the meta-analysis. Abstracts, reviews, case reports and articles which did not show efficient information were excluded. If multiple studies were published on the same population or subpopulation, the most recent or informative one would be included in the meta-analysis. The flow chart of the selection of publications included in the meta-analysis is shown in Figure 1. The quality assessment of included studies was further conducted according to the Cochrane handbook 5.1.0 for random control trial (RCT) (http://www.cochrane.org/training/cochrane-handbook) and Newcastle-Ottawa Scale (NOS) for non-randomized study such as cohort study and case-control study (http://www.ohri.ca/programs/clinical_epidemiology/oxford.asp).

### Data extraction

Information was extracted from all eligible publications independently by two of the authors (Yanwen Yao and Xiaoling Gu). The items included in the data form were as follows: study name (together with the first author's name and year of publication), country, study type, study population, age of the subjects, type of used hormone, study-specific ORs or RRs with 95% CI, detailed numbers of patients for “non-HRT user versus HRT user” including “the short period use versus the long period use” and matched or adjusted variables in the analysis. For studies from different countries, the ethnicity of subjects was also collected. Most of the estimated associations between HRT use and lung cancer were adjusted for some factors or their combination. According to the suggestion from Chen et al [10], if both the univariate OR/RR and multivariate-adjusted OR/RR were provided, multivariate-adjusted OR/RR was extracted. The lists from the authors were compared, and disagreements were resolved by consensus.

### Statistical analyses

OR/RR and corresponding 95%CI or detailed numbers of patients in each included study was pooled to evaluate the association between HRT and lung cancer risk. Whenever available, we used multivariate-adjusted risk estimates; otherwise, we utilized or computed distribution given in the papers the unadjusted ORs. Since not all including studies providing ORs/RRs but all the detailed numbers of patients were available, we entered the number of patients with HRT use, non-HRT use, lung cancer patients in HRT users and lung cancer patients in non-HRT users to total meta-analysis. Stratified analyses were also carried out by study type, BMI status, histology, smoking status, and menopause type.

Heterogeneity assumption was evaluated with a chi-square-based Q-test. The summary OR estimate of each study was calculated by a fixed effects model (the Mantel-Haenszel method). If the *P* value is greater than 0.05 of the Q-test, which indicates low heterogeneity or lack of heterogeneity among the included studies, the fixed effects model is proper, otherwise, the random-effects model (the DerSimoniane and Laird method) was performed [11], [12]. Sensitive analysis was also performed to evaluate the influence of individual studies on the final effect. Potential publication bias would be observed by the funnel plot and formally evaluated with Begg's adjusted rank correlation test and Egger's linear regression test. All statistical analysis were done with the Stata software (version 11.0; StataCorp LP, College Station, TX, USA), using two-sided *P* values.

## Results

### Study selection and characteristic in the meta-analysis

A total of 25 eligible studies met the inclusion criteria and entered into the final meta-analysis [4]–[9], [13]–[31]. All studies passed the quality assessment and the result was shown in Table 1. The published years of studies, study types, regions, ethnicity of included patients, the number of patients, the mean age or age range of patients, and type of HRT were all collected. The characteristics of selected studies are presented in Table 2. The time range of studies was from 1988 to 2012. The total number of participates was 656,403 and final 11,442 participates were histologically diagnosed to be lung cancer patients. Four studies were random control trials (RCTs) and ten were case-control studies. Among the total 25 studies, two studies were conducted in the Asian females [7], [18], five studies were in Caucasian [17], [20]–[22], [26] and the ethnicity in the left studies were mixed or not given. Females in studies of Blackman et al., Kabat et al., Liu et al., Olsson et al. and Smith et al. included young females, who were less than 50 years old [13], [16], [18], [20], [27]. Estrogen was used alone as HRT in seven studies [4], [5], [14], [20], [28], [30], [31], estrogen plus progestin were used as HRT in three studies [6], [15], [24] and estrogen or estrogen plus progestin or the combination of both was used in the left 15 studies. The status of oral contraceptive use in patients was also investigated in nine studies [9], [14], [16], [17], [23], [27]–[29], [31].

**Table 1:** Quality assessment scores of the cohort and case-control studies.

Study	Study type	Selection	Comparability	Outcome
Adami[4]	Cohort	4	2	3
Brinton[30]	Cohort	4	2	3
Clague[8]	Cohort	2	1	3
Kabat[16]	Cohort	4	2	3
Liu[18]	Cohort	3	2	3
Olsson[20]	Cohort	3	2	3
Persson[21]	Cohort	4	2	3
Pukkala[22]	Cohort	4	2	3
Rodriguez[23]	Cohort	3	2	3
Slatore[26]	Cohort	4	2	3
Smith[27]	Cohort	4	2	3

Study	Study type	Selection	Comparability	Exposure
Blackman[13]	Case-control	4	1	2
Chen[7]	Case-control	4	2	3
Elliott[14]	Case-control	4	2	3
Kreuzer[17]	Case-control	4	2	3
Mahabir[19]	Case-control	4	2	2
Meinhold[31]	Case-control	4	2	3
Ramnath[9]	Case-control	3	2	3
Schabath[25]	Case-control	4	2	3
Taioli[28]	Case-control	3	2	3
Wu[29]	Case-control	4	2	3

RCT	Chlebowski[6]	Chlebowski[5]	Hulley[15]	RossouwJE[24]
Adequate sequence generation	Y	Y	Y	Y
Allocation concealment	Y	Y	Y	Y
Blinding	Y	Y	Y	Y
Incomplete outcome data addressed	NA	Y	Y	Y
Free of seletive reporting	Y	Y	Y	Y
Free of other bias	Y	Y	Y	Y

**Table 2:** Baseline information of the 25 included studies in meta-analysis.

Study	Year	Region	Ethnicity	Study type	Participants	Cases	Age(years)	Hormone Type		
Adami[4]	1989	US	NG	Cohort	23244	82	54.5		E	
Blackman[13]	2002	US	Mixed	Case-control	4188	659	40–74		E	E+P
Brinton[30]	2012	US	Mixed	Cohort	118008	2541	64.6		E	E+P
Chen[7]	2007	China	Asian	Case-control	1357	826	57		E	E+P
Chlebowski[6]	2009	US	Mixed	RCT	16608	194	50–79			E+P
Chlebowski[5]	2010	US	Mixed	RCT	10739	115	50–79		E	
Clague[8]	2011	US	Mixed	Cohort	60592	727	50–77		E	E+P
Elliott[14]	2006	Scotland	NG	Case-control	648	162	NG	O	E	
Hulley[15]	2010	US	Mixed	RCT	2763	64	69			E+P
Kabat[16]	2007	Canada	NG	Cohort	89812	750	40–59	O	All	
Kreuzer[17]	2003	Germany	Caucasian	Case-control	1723	800	<76	O	All	
Liu[18]	2005	Japan	Asian	Cohort	44677	137	40–69		All	
Mahabir[19]	2008	US	Mixed	Case-control	1601	763	60		All	
Meinhold[31]	2011	US	NG	Case-control	1041	430	66	O	E	E+P
Olsson[20]	2003	Sweden	Caucasian	Cohort	29508	55	25–65		All	
Persson[21]	1996	Sweden	Caucasian	Cohort	22597	223	54.5		E	
Pukkala[22]	2001	Finland	Caucasian	Cohort	94505	120	NG		All	
Ramnath[9]	2007	US	Mixed	Case-control	1790	595	61	O	All	
Rodriguez[23]	2008	US	NG	Cohort	72772	659	NG	O	All	
RossouwJE[24]	2002	US	Mixed	RCT	16608	104	63			E+P
Schabath[25]	2004	US	Mixed	Case-control	1018	499	NG		All	
Slatore[26]	2010	US	Caucasian	Cohort	36588	334	50–76		E	E+P
Smith[27]	2009	US	NG	Cohort	2861	87	31–79	O	All	
Taioli[28]	1994	US	NG	Case-control	483	180	NG	O	E	
Wu[29]	1988	US	NG	Case-control	672	336	59	O	E	E+P

RCT: random clinical trial, O: oral contraceptive use, E: estrogen replacement, P: progestin replacement, E+P: estrogen and progestin combination therapy, NG: Not Given or No limitation.

### Meta analyses on HRT use and lung cancer risk

ORs and 95% CI were not all demonstrated in the included 25 studies. However, the number of lung cancer patients in HRT users and in non-HRT users were provided in all the studies, so the detailed numbers in the two groups were collected and shown in Table 3. In subgroup analyses, ORs and the corresponding 95% CIs were collected and meta-analyses were performed in different subgroups.

**Table 3:** Numbers of lung cancer patients in HRT and non-HRT groups from25 studies.

	HRT user		Non-HRT user			
Studies	Lung Cancer	Total	Lung Cancer	Total	OR	95%CI
Adami[4]	46	23244	36	23244	1.278	0.826–1.978
Blackman[13]	145	762	514	2767	1.030	0.839–1.264
Brinton[30]	1002	67137	1539	45443	0.880	0.812–0.953
Chen[7]	145	279	681	1078	0.631	0.484–0.823
Chlebowski[6]	109	8506	85	8102	1.224	0.920–1.628
Chlebowski[5]	61	5310	54	5429	1.157	0.800–1.672
Clague[8]	511	45187	216	15405	0.804	0.685–0.944
Elliott[14]	19	74	143	574	1.041	0.598–1.813
Hulley[15]	37	1380	27	1383	1.384	0.838–2.285
Kabat[16]	274	42156	476	47656	0.648	0.559–0.753
Kreuzer[17]	196	470	604	1236	0.748	0.604–0.928
Liu[18]	24	5276	113	36077	1.454	0.935–2.262
Mahabir[19]	298	692	465	909	0.722	0.592–0.881
Meinhold[31]	194	505	236	536	0.793	0.619–1.016
Olsson[20]	8	3040	47	25515	1.430	0.675–3.028
Persson[21]	112	22597	111	22597	1.009	0.776–1.313
Pukkala[22]	55	94505	65	94505	0.846	0.591–1.212
Ramnath[9]	132	470	463	1320	0.723	0.574–0.910
Rodriguez[23]	355	40013	304	32759	0.956	0.819–1.115
RossouwJE[24]	54	8506	50	8102	1.029	0.699–1.513
Schabath[25]	232	505	267	513	0.783	0.612–1.002
Slatore[26]	230	23119	104	11642	1.115	0.883–1.407
Smith[27]	33	1024	54	1837	1.100	0.708–1.707
Taioli[28]	62	138	118	345	1.569	1.049–2.347
Wu[29]	149	336	187	336	0.635	0.468–0.861

### Test of heterogeneity

Heterogeneity between studies was observed regarding both overall comparisons and subgroup analyses. According to the I^2^ and *P* for heterogeneity test listed in the Table 4, the random effect model which based on the Mantel-Haenszel method was adopted when I^2^ ≥50% and *P* for heterogeneity test ≤0.05. Otherwise, the fixed effect model was adopted.

**Table 4:** Stratified analyses of Hormone therapy on lung cancer risk.

			Heterogeneity			
Subgroup	N	Model	*I^2^*(%)	*P*	OR(95%CI)	*P*
All Studies	25	Random	68.	0.000	0.90(0.82–0.98)	0.033
Case-control studies	10	Random	63.9	0.003	0.81(0.70–0.93)	0.002
Cohort Studies	11	Random	70.4	0.000	0.94(0.83–1.06)	0.318
Random Control studies	4	Fixed	0.0	0.814	1.18(0.99–1.42)	0.073
BMI<25 kg/m^2^	4	Fixed	30.0	0.407	0.65(0.53–0.79)	0.000
25≤BMI<30 kg/m^2^	4	Fixed	25.5	0.258	0.84(0.65–1.09)	0.188
BMI≥30 kg/m^2^	4	Fixed	0.0	0.434	0.89(0.63–1.26)	0.514
NSCLC	6	Random	58.1	0.036	0.93(0.76–1.14)	0.506
SCLC	6	Fixed	0.0	0.663	1.05(0.83–1.33)	0.698
Adenocarcinoma	9	Random	50.6	0.040	0.96(0.82–1.11)	0.573
Never Smokers	11	Fixed	0.0	0.502	0.86(0.75–0.99)	0.042
Smokers	10	Random	52.8	0.025	0.94(0.82–1.07)	0.333
Oral Contraceptive	7	Random	54.2	0.041	0.97(0.80–1.18)	0.772
Age at Menopause>52	4	Random	62.0	0.048	0.84(0.60–1.17)	0.297
Artificial Menopause	4	Fixed	0.0	0.419	1.51(1.17–1.94)	0.001
Duration of HRT use≤5 years	5	Fixed	0.0	0.553	0.98(0.87–1.10)	0.709
Duration of HRT use>5 years	5	Fixed	20.5	0.284	0.99(0.87–1.14)	0.919

As shown in Table 4, *P* for heterogeneity test of lung cancer patients in HRT users vs. non-HRT users for all studies was 0.000 and I^2^ was 67.6%, which meant there was heterogeneity for the meta-analysis, so the random effect model was adopted. In the subgroup analyses, meta-analyses on RCTs, BMI<25 kg/m^2^, 25≤BMI<30 kg/m^2^, BMI≥30 kg/m^2^, small cell lung cancer (SCLC), never smokers, artificial menopause, duration of HRT use≤5 years and duration of HRT use>5 years were performed on the fixed effect model, other subgroup analyses were used random effect models.

### Meta-analysis results

**Figure 2:**
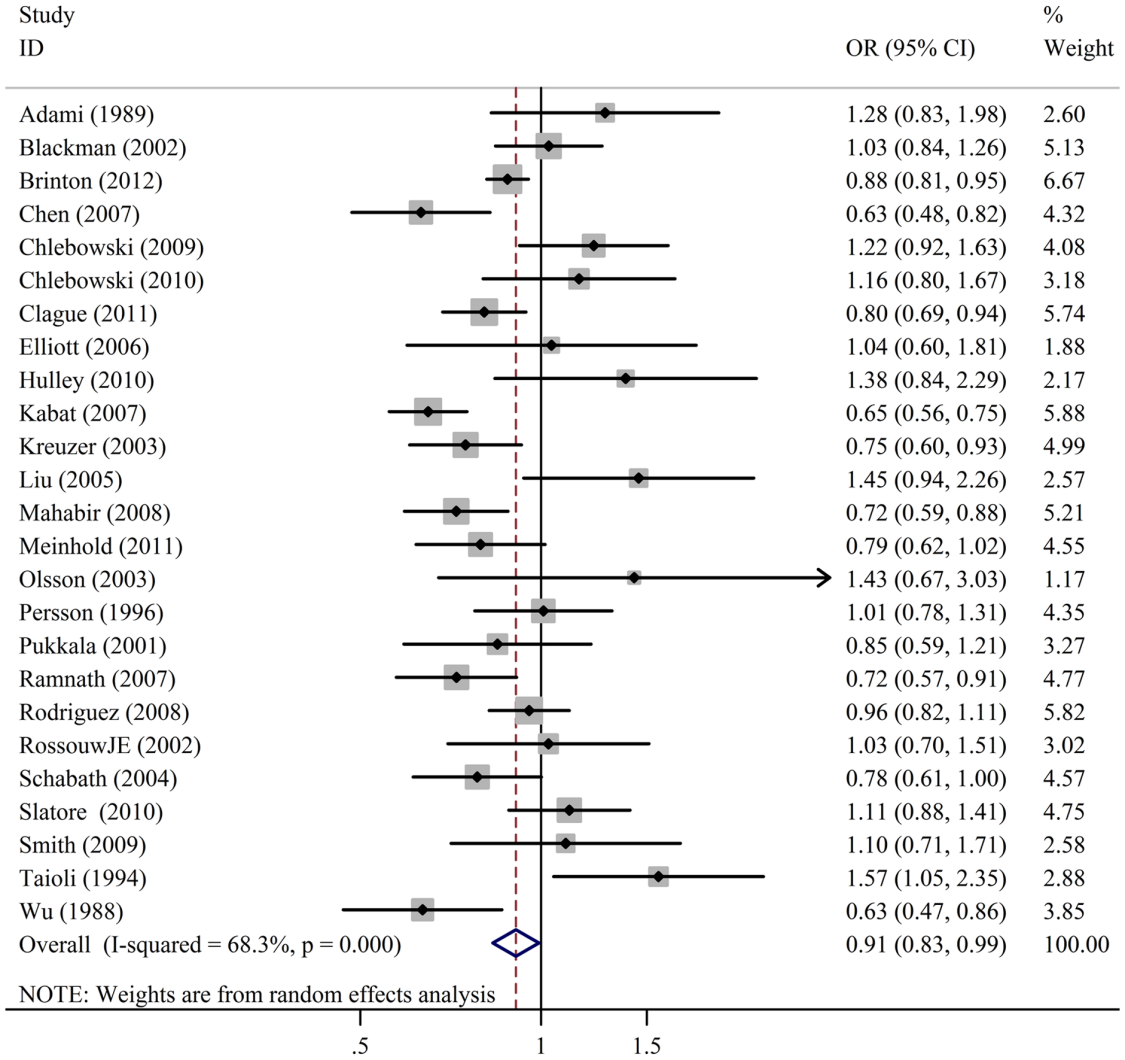
Meta-analysis with a random-effects model for the association between HRT use and cancer risk in females. CI: confidence interval.

**Figure 3:**
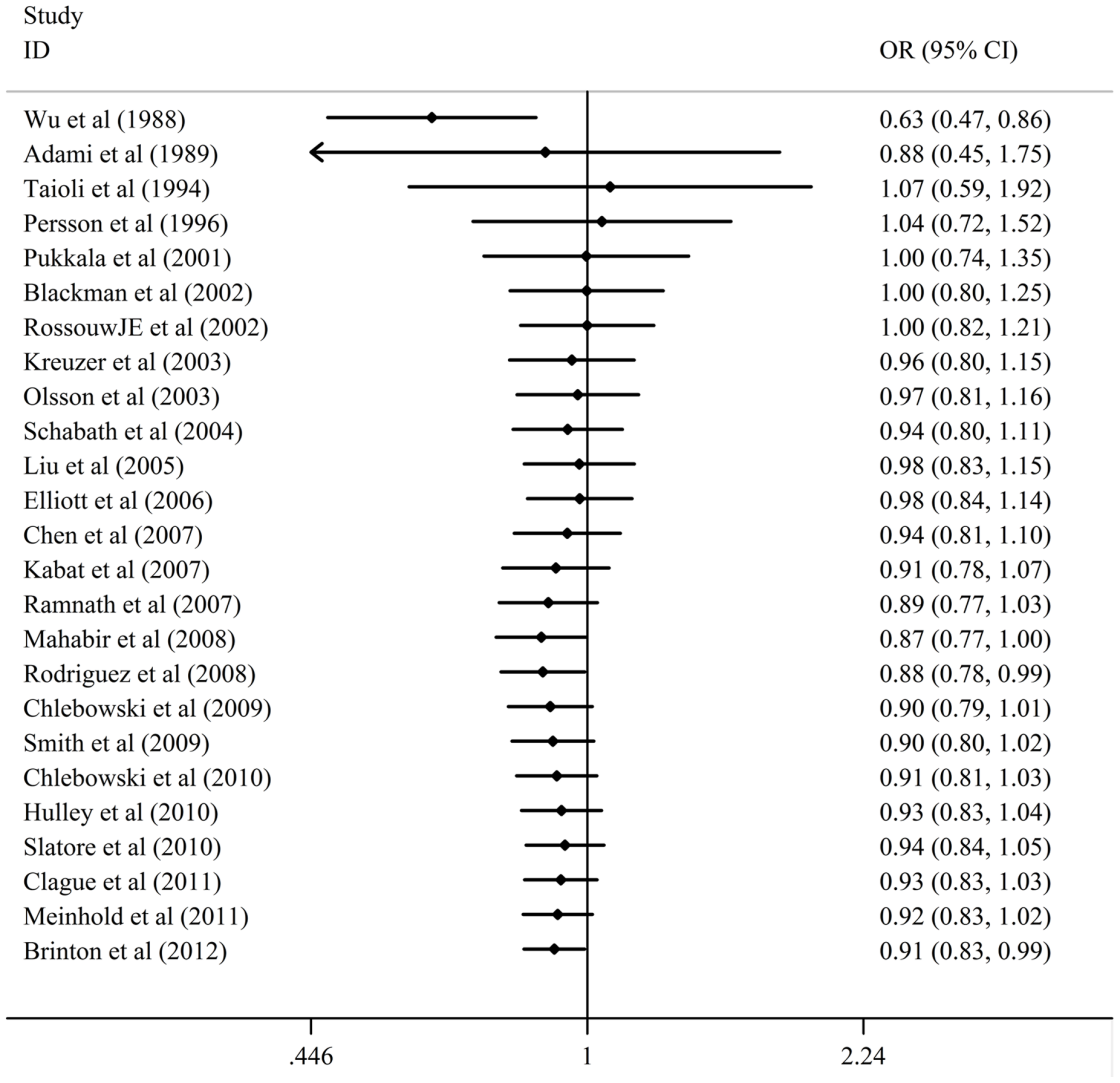
A cumulative meta-analysis tracked over time with a random-effects model for the association between HRT use and cancer risk in females. CI: confidence interval.

The result of the meta-analysis on the all 25 studies and the forest plot was shown in Table 3 and Figure 2. OR was 0.91(95%CI  = 0.83 to 0.99) and *P* value of the test for overall effect was 0.033 when analysis performed using random effect model. This result suggested that HRT use was associated with a significant decrease risk of lung cancer compared with non-HRT use in females. A cumulative meta-analysis was further conducted to confirm the result. As shown in Figure 3, the result was shifted to an association between HRT use and a significant decrease risk of lung cancer when the evidence was tracked over time.

**Figure 4:**
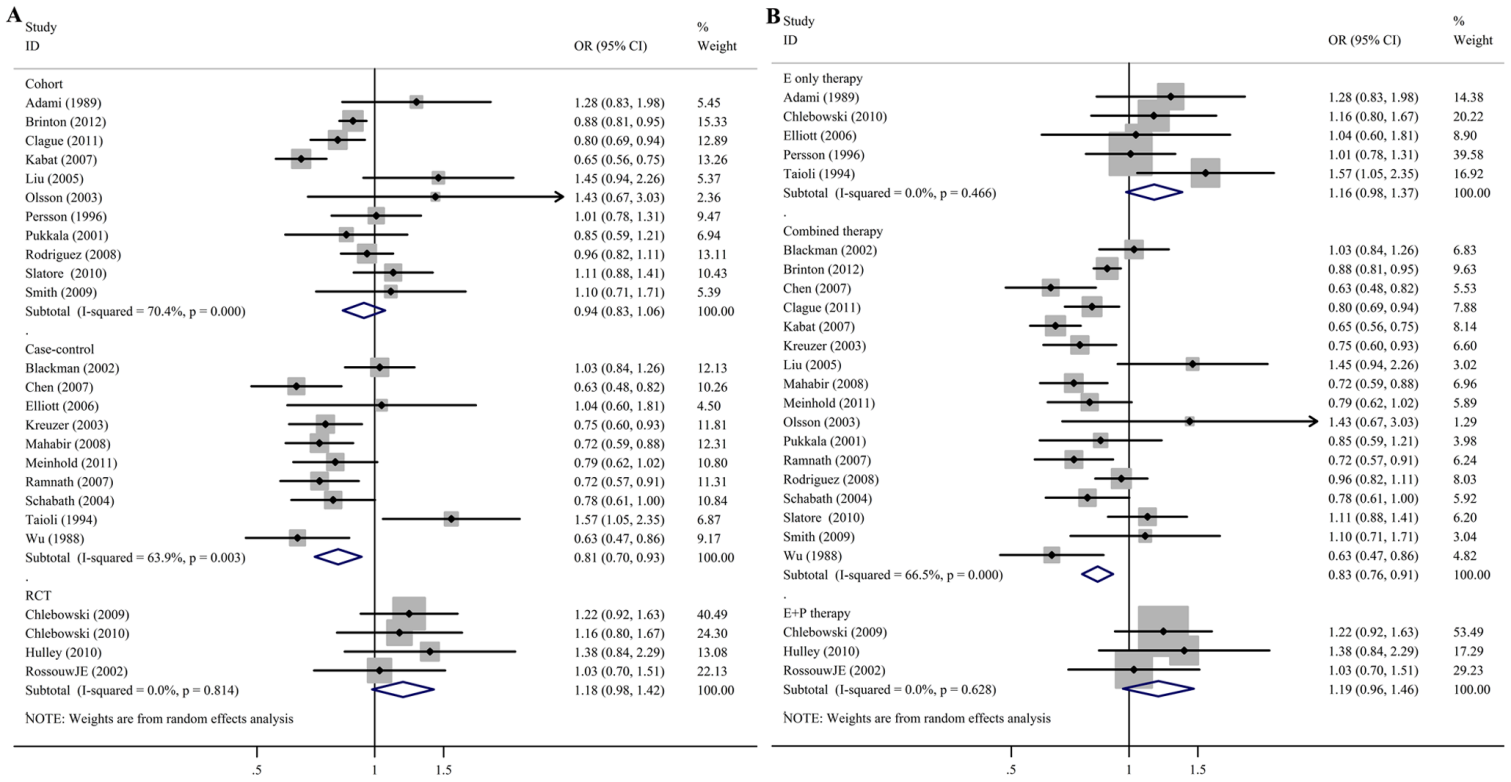
A: Meta-analysis with a random-effects model for the association between HRT use and cancer risk in females stratified by study type. B: Meta-analysis with a random-effects model for the association between HRT use and cancer risk in females stratified by hormone type. CI: confidence interval.

Since heterogeneity was revealed in the overall meta-analysis, subgroup analyses were performed after stratifications of data by study type, BMI status, histology, smoking status, oral contraceptive use, menopause type, and duration of HRT use. The overall ORs and corresponding 95% CI of subgroup analyses were demonstrated in Table 4. In analysis of ten case-control studies, the association between significant decreased lung cancer risk and HRT use was also been revealed, while increased lung cancer risk was correlated with HRT use according to the meta-analysis of four RCTs but the difference is not significant. The forest plot of the study type subgroup analysis is demonstrated in Figure 4–A. In the analysis of different types of hormone replacement therapy, estrogen, progestin or the combination of both were used in participates in 17 studies. The sub-analysis result of these 17 studies revealed that this type of hormone use could reduce the lung cancer risk (OR = 0.83, 95%CI 0.76–0.91, P = 0.000) (Figure 4–B). Five studies in which estrogen was used as the replacement hormone demonstrated that although the difference was not significant, only estrogen use could increase the lung cancer risk (OR = 1.16, 95%CI 0.98–1.37, P = 0.079).

**Figure 5:**
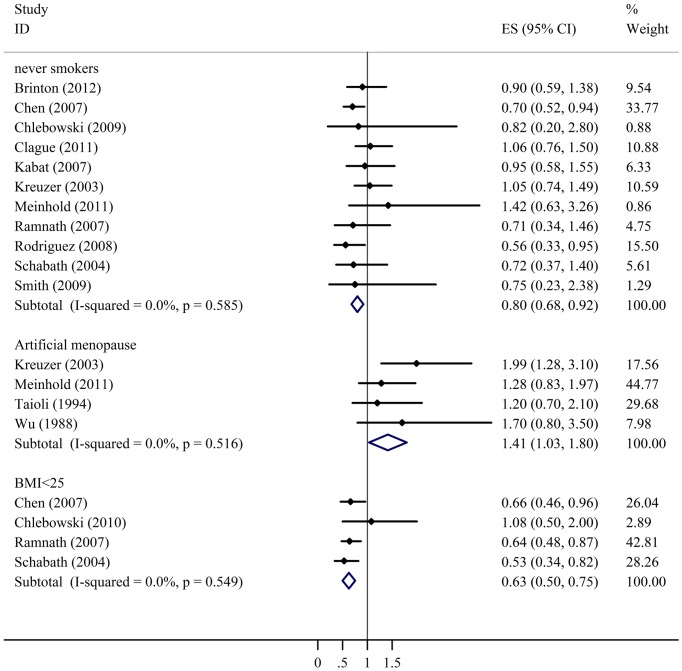
Meta-analysis for the significant association between cancer risk and HRT use in females with artificial menopause or BMI<25 kg/m^2^ or non-smoking females. CI: confidence interval.

As shown in Table 4 and Figure 5, the positive association between HRT use and decreased lung cancer risk was found in the patients with never smokers patients (OR = 0.86, 95%CI 0.75–0.99, *P* = 0.042), and BMI<25 kg/m^2^ (OR = 0.65, 95%CI 0.53–0.79, *P* = 0.000). However, HRT use in patients with artificial menopause increased the lung cancer risk (OR = 1.51, 95%CI 1.17–1.94, *P* = 0.001). A subgroup analysis was also done on HRT use in smokers, but the evidence showed negative correlation between smoking and the risk of lung cancer, *P* = 0.333.

### Publication bias

**Figure 6:**
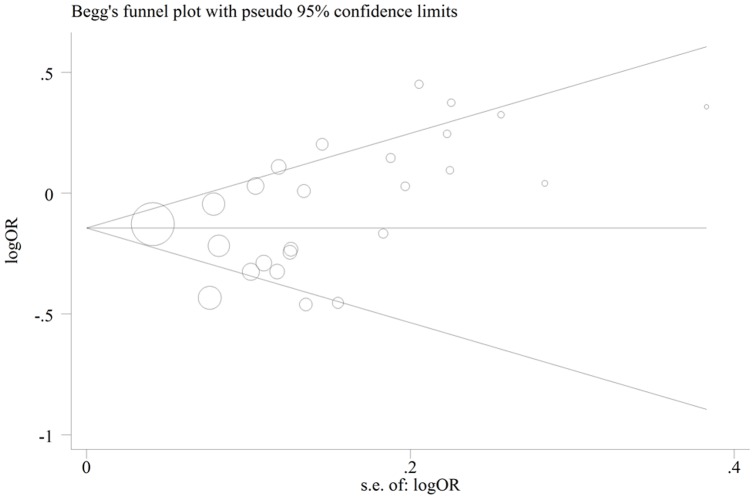
Begg's funnel plot of publication bias test. Each point represents a separate study for the indicated association. Log (OR): natural logarithm of OR; Horizontal line: mean effect size.

The publication bias of the literatures was assessed by using Begg's funnel plot and Egger's test. The shape of the funnel plots seemed approximately symmetrical (Figure 6) and the result of Egger's test did not show any evidence of publican bias, t = 1.91 and *P* = 0.069 for HRT use vs. non-HRT use.

### Sensitivity analysis

**Figure 7:**
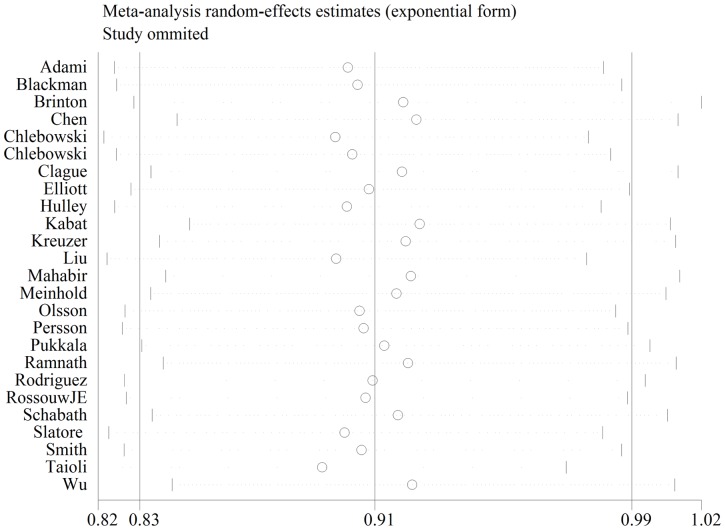
Sensitivity analysis of the included 25 studies. Each point represents a OR and corresponding 95%CIs after excluding each study.

Sensitivity analysis was also analyzed as previous study (Table 5 and Figure 7) [32]. As shown in Figure 7, none of the studies appears to be an outlier or has results very different from the rest of the studies. After each study was excluded from the overall meta-analysis, the similar results were obtained, which suggested that the result of the meta-analysis was stable.

**Table 5:** Sensitivity analysis: ORs, corresponding 95%CIs and *P* values after excluding each study.

Excluded Study	OR	95%CI	*P* value
Adami[4]	0.899	0.823–0.983	0.019
Blackman[13]	0.902	0.823–0.989	0.028
Brinton[30]	0.917	0.829–1.015	0.095
Chen[7]	0.922	0.843–1.007	0.072
Chlebowski[6]	0.895	0.819–0.978	0.014
Chlebowski[5]	0.901	0.823–0.985	0.022
Clague[8]	0.917	0.835–1.007	0.071
Elliott[14]	0.906	0.828–0.991	0.032
Hulley[15]	0.899	0.823–0.982	0.018
Kabat[16]	0.923	0.847–1.005	0.064
Kreuzer[17]	0.918	0.837–1.007	0.069
Liu[18]	0.895	0.820–0.977	0.013
Mahabir[19]	0.920	0.839–1.008	0.073
Meinhold[31]	0.915	0.834–1.003	0.059
Olsson[20]	0.903	0.826–0.987	0.024
Persson[21]	0.904	0.825–0.991	0.031
Pukkala[22]	0.911	0.832–0.998	0.045
Ramnath[9]	0.919	0.839–1.007	0.069
Rodriguez[23]	0.907	0.826–0.997	0.042
RossouwJE[24]	0.905	0.827–0.991	0.031
Schabath[25]	0.916	0.835–1.004	0.061
Slatore[26]	0.898	0.821–0.983	0.019
Smith[27]	0.904	0.826–0.989	0.028
Taioli[28]	0.891	0.817–0.971	0.008
Wu[29]	0.920	0.842–1.006	0.068

## Discussion

To date, the current and estimated morbidity and mortality of lung cancer in females are still less than that in males [2]. Although much has been learned about the epidemiology, the reason is still unclear. Besides the different smoking status, the kind and altering levels of endogenous and exogenous hormone in males and females has become to be the subject of the epidemiology study.

As the most common therapy for menopausal females and young females with artificial menopause, HRT has been proved to be associated with increased risk of breast cancer and endometrial cancer and decreased risk of colorectal cancer and coronary heart disease [33]–[37]. The role of HRT in lung cancer incidence has also been investigated in several studies. Wu et al in their study firstly demonstrated that the menopause type and hormone intake were associated with the lung cancer risk in female [29]. Previous studies in past years revealed the controversial association between HRT and lung cancer risk.

Our meta-analysis of total 25 studies on HRT use and lung cancer risk demonstrated that HRT use in female could decrease the risk of lung cancer. The positive correlation was also identified between decreased lung cancer risk and HRT use in females with BMI<25 kg/m^2^ and never smokers, increased lung cancer risk and HRT users with artificial menopause.

Estrogen in HRT was considered to be the most likely candidate for mediating growth-promoting effect in lung cancer, because evidence showed that expression of estrogen receptors (ER) in lung cancer mediated transcriptional responses in lung cancer cells [38]–[40]. On the other side, progestin which is also a type of hormone in HRT is reported to take a protect role against lung cancer [40], [41]. In the study of Ishibashi, progestin receptor was revealed to be in about half of lung cancer, and progestin mediated pathways to induce apoptosis and reduce growth in lung cancer cells [41]. These results demonstrated that HRT can act tumor growth-promoting or tumor growth-suppressing effects depending on the components of HRT, the expressions of estrogen receptors and progestin receptors.

Moore et al firstly suggested in his study that according to the respective data, young women (age from 31 to 50 years) presented more often with advanced disease [42]. This may help to explain why HRT users with artificial menopause who were always young women had increased lung cancer risk. The mechanism involved may be estrogen plays a deleterious role in lung cancer in younger female patients, especially in early stage disease [42]. The reason for undefined risk in smoking HRT users may be that higher circulating levels of estrogen in women make susceptible to the carcinogenic influence of tobacco smoke [43].

The analyses showed that the ORs and significance were different in case control studies, cohort studies and RCT. As provided in each article, all females in four RCT studies included in this meta-analysis, were postmenopausal women, with the age range or the mean age of 50–79, 50–79, 69 and 63, respectively. While in cohort and case-control studies, females were younger than those in RCTs, suggesting these females were not in the menopause or were close to the menopause.

Vandenbroucke JP [44] indicated that adjustment for previous use of hormones already increased the estimates in the trials. HRT is usually started close to menopause, so the observational studies had shown a relatively true situation. However, this situation was diluted in the randomized trial because fewer women close to menopause were enrolled. The discrepancy between the RCT and observational study was not only in HRT and lung cancer, but also in HRT and coronary heart disease, and breast cancer. It was not due to differences in study design, but to the timing of start of treatment.

Randomized trials are necessary for showing whether the benefit of a medical intervention exists [45]. In contrast, observational study is often used to investigate adverse effects [46]. It was suggested that the same adverse effect for the same treatment can rarely be investigated by observational research and in very large randomized trials [47], such as breast cancer and HRT, and lung cancer and HRT shown in our meta-analysis. Vandenbroucke JP also suggested that observational studies may better reflect the true harm in real-life than selected populations in randomized trials [44].

In the subgroup analyses HRT use was confirmed to be associated with decreased lung cancer risk in non-smoking females. However, HRT use in ever smoking females failed to be associated with lung cancer risk. The fact that smoking can induced increased lung cancer risk in females could help to explain the result. In a study on smoking-related mortality in the United States, smoking was shown to be also hazardous for women comparable to men [48]. Since smoking and other socio-economic status were proved to be associated with increased lung cancer risk, the effect of HRT may be potentially confounding by these factors.

The present study provides a quantitative analysis of available epidemiologic studies on HRT and lung cancer risk in females. There is a broad time span in the 25 studies, from 1988 to 2012, and the sample size is large (total 656,403 participates). Stratified analyses were conducted. Heterogeneity was tested and random-effects model was used to estimate the magnitude of the heterogeneity and assign a greater variability to the combined risk estimate to account for the heterogeneity if detected. Publication bias was also examined and corrected if detected.

Some limitations of this meta-analysis should be acknowledged. First, many observational studies which were susceptible to various biases were included in our meta-analyses. The subgroup analysis in different types of studies showed different results. However, taking these results together still shows the positive association between HRT and lung cancer risk. Second, although subgroup analyses was proceed by the kind of HRT, HRT dose which may also affects the result were not taking into analysis. Finally, only published studies were included in our meta-analysis. Therefore, publication bias may be occurred although no publication bias was indicated from both funnel plot and Egger's test.

### Conclusion

Taken together, our analysis of currently available studies on HRT and lung cancer risk suggests that HRT use is correlated with decreased lung cancer risk in female, especially in female with BMI<25 kg/m^2^ and never smokers, but HRT in female with artificial menopause could increase the lung cancer risk. Considering that lung cancer is one of the leading causes of death in female, further investigations or larger observational study with women closer to menopause rather than postmenopausal was needed in future to further validate the influence of HRT in lung cancer.

## Supporting Information

Checklist S1 (DOC)
